# A Novel HMM Distributed Classifier for the Detection of Gait Phases by Means of a Wearable Inertial Sensor Network

**DOI:** 10.3390/s140916212

**Published:** 2014-09-02

**Authors:** Juri Taborri, Stefano Rossi, Eduardo Palermo, Fabrizio Patanè, Paolo Cappa

**Affiliations:** 1 Department of Mechanical and Aerospace Engineering, Sapienza University of Rome, Via Eudossiana 18, I-00184 Roma, Italy; E-Mails: j.taborri@gmail.com (J.T.); eduardo.palermo@uniroma1.it (E.P.); paolo.cappa@uniroma1.it (P.C.); 2 Department of Economics and Management, Industrial Engineering (DEIM), University of Tuscia, Via del Paradiso 47, I-01100 Viterbo, Italy; 3 School of Mechanical Engineering, Niccolò Cusano University, Via Don Carlo Gnocchi 3, I-00166 Roma, Italy; E-Mail: fabrizio.patane@unicusano.it; 4 MARLab, Movement Analysis and Robotics Laboratory, Neurorehabilitation Division, IRCCS Children's Hospital “Bambino Gesù”, Via Torre di Palidoro, I-00050 Fiumicino (RM), Italy

**Keywords:** gait detection, Hidden Markov Models, hierarchical decision, distributed classifier, wearable sensor network, gyroscopes

## Abstract

In this work, we decided to apply a hierarchical weighted decision, proposed and used in other research fields, for the recognition of gait phases. The developed and validated novel distributed classifier is based on hierarchical weighted decision from outputs of scalar Hidden Markov Models (HMM) applied to angular velocities of foot, shank, and thigh. The angular velocities of ten healthy subjects were acquired via three uni-axial gyroscopes embedded in inertial measurement units (IMUs) during one walking task, repeated three times, on a treadmill. After validating the novel distributed classifier and scalar and vectorial classifiers-already proposed in the literature, with a cross-validation, classifiers were compared for sensitivity, specificity, and computational load for all combinations of the three targeted anatomical segments. Moreover, the performance of the novel distributed classifier in the estimation of gait variability in terms of mean time and coefficient of variation was evaluated. The highest values of specificity and sensitivity (>0.98) for the three classifiers examined here were obtained when the angular velocity of the foot was processed. Distributed and vectorial classifiers reached acceptable values (>0.95) when the angular velocity of shank and thigh were analyzed. Distributed and scalar classifiers showed values of computational load about 100 times lower than the one obtained with the vectorial classifier. In addition, distributed classifiers showed an excellent reliability for the evaluation of mean time and a good/excellent reliability for the coefficient of variation. In conclusion, due to the better performance and the small value of computational load, the here proposed novel distributed classifier can be implemented in the real-time application of gait phases recognition, such as to evaluate gait variability in patients or to control active orthoses for the recovery of mobility of lower limb joints.

## 1. Introduction

The identification of events and phases in the human gait is an essential starting point for: (i) assessing the degree of recovery in walking ability in patients after interventions or rehabilitation treatments [1,2]; (ii) classifying the activity of daily living, including the overall health status of individuals [3,4]; and (iii) controlling synchronously active orthoses and exoskeletons for the recovery of lower limb mobility [5].

Several approaches and technologies have been developed in order to detect gait phases. Motion capture systems based on marker tracking and six-component force platforms still represent the gold standard for extracting gait patterns [6–10]. Alternatively, the detection of human motion can be evaluated by means of self-contained wearable systems, which do not rely on camera-based systems and can be also used outdoors for continuous data logging. The most used sensors are: wireless pressure sensing shoe insoles [11,12], shoe-mounted foot switches [13], smart textiles [14], accelerometers [15], gyroscopes [5,16,17], and the composite inertial measurement unit system IMU [18,19]. The cited references represent a few of the numerous works available in the literature.

Computational methodologies for gait phase detection fall into two major categories. Firstly, the post-processing analysis uses algorithms which partition the gait phases through the threshold selection of raw data [20–22]. Secondly, the procedure is based on matching-learning schemes which extract patterns on the basis of Support Vector Machines (SVM) [23], Linear Discriminant Analysis (LDA) [24], Gaussian Mixture Model (GMM) [25], and, finally, Hidden Markov Model (HMM) [5,16,26,27]; this procedure recently received a great deal of interest for its potential better performance. From a comparative examination of the matching-learning schemes, Mannini and Sabatini [26] have shown higher performance for HMM than SVM, GMM, and LDA in the recognition of seven different motor activities, using a classification method based on the analysis of outputs from four tri-axial linear accelerometers placed on hip, wrist, ankle, and thigh. The authors demonstrated that HMM is characterized by the highest values of accuracy, sensitivity and specificity. Abaid *et al.* [5] proved the applicability of a scalar HMM based on data from a uni-axial gyroscope to control powered orthoses worn by children, testing the HMM both in either normally developed children or children with hemiplegia. The algorithm proposed by the authors showed a sensitivity and specificity higher than 0.95 in the detection of gait phases.

In the case of a sensor network, hierarchical classifiers based on data-fusion processing were implemented after matching-learning algorithms in order to improve the performance [28] and robustness [29] of the classifiers. Kittler and colleagues [30] examined two methods of post-processing data based on the implementation of a hierarchical classification: Hierarchical Decision (HD) and Majority Voting (MV). The HD is based on giving more importance to the simple classifiers and then letting them decide first, due to their overall better performance. In the MV, however, the same opportunity is given to all the classifiers and the final decision is the one which obtains more votes. Briefly, the main limitations are: the HD is used only with a few decision classifiers; the MW is dependent on the effects of noisy environments, where a minority of high-accuracy decision entities can be hidden by a majority of weak-decision ones [28]. To overcome the above-mentioned limitations, several studies, in different research fields [28,31–35], introduced a Hierarchical Weighted Decision (HWD). HWD gives to all the entities the opportunity to collaborate in the decision making, while ranking the relative importance of each decision through the use of weights based on the individual performance of each entity. In particular, Banos *et al.* [28] demonstrated that the use of a hierarchical weighted decision allows to take the right decision in more cases with respect to HD and MV. However, this technique has not been applied, to the authors' knowledge, to detect gait phases.

From this perspective, we propose a novel HWD algorithm, called “Distributed Classifier” (DC), for the detection of gait phases in real-time applications. DC is based on the processing, by means of HMM, of two or more outputs gathered by uni-axial gyroscopes placed on different body segments of lower limbs. The goals of this study are two-fold. Firstly, we seek to answer the question of whether the DC can be used to detect gait phases. Secondly, we investigate whether the DC can be implemented in real-time applications, such as the control of active orthoses for the recovery of the mobility of lower limb joints. To validate the novel classifier we computed sensitivity, specificity and computational load and comparatively examined them with the values obtained with two classifiers based on scalar (SC) and vectorial (VC) HMM [36] that were already proposed and validated in gait detection.

## 2. Material and Methods

### 2.1. Theoretical Approach

In the present work, a new gait phase distributed classifier (DC), based on a Hidden Markov Model (HMM), is analyzed. In order to describe the DC, a short description of the scalar and vectorial HMM classifiers (SC and VC, respectively) follows.

#### 2.1.1. Hidden Markov Model

HMM is a powerful statistical model for classification of time series [37,38]. HMM is defined as a doubly embedded stochastic process with an underlying process that is not observable, *i.e.*, it is hidden. The hidden process can only be observed through another set of stochastic processes that produce the sequence of observations [36].

The set of the *Q* model states, and the set of the actual *N* model states as a function of time are, respectively:

(1) $$\begin{array}{cc} {\left\{S_{i}\right\}}_{Qx1} & {\left\{X_{i}\right\}}_{Nx1} \end{array}$$

The observation **Y** at time *t_n_* is thevector of *M* acquired signals which are emitted by the current state output at time*t_n_*. The vector **Y** is:

(2) $$\text{Y}\left(t_{n}\right)=\left\{Y_{1}\left(t_{n}\right)\ldots Y_{M}\left(t_{n}\right)\right\}$$

HMM can be written as a set **λ** of three parameters **A**, **B**, and **π**:

(3) λ=(A,B,π)

where:

- **A** is the probability distribution matrix of the state transitions:
  (4) $$\begin{array}{cc} \text{A}={\left\{a_{ij}\right\}}_{QxQ} & a_{ij} \end{array}=\text{P}\left(X\left(t_{n}\right)=S_{i}|X\left(t_{n+1}\right)=S_{j}\right)$$
- **B** is the probability distribution matrix associated with the *M*-size set of the *Y* observations at the state *S_i_*:
  (5) $$\begin{array}{cc} \text{B}={\left\{b_{ij}\right\}}_{QxN} & b_{ij} \end{array}=\text{P}\left(Y\left(t_{n}\right)=Y_{j}|X\left(t_{n}\right)=S_{i}\right)$$
  Moving to a continuous HMM (cHMM), the previous probability can be computed hypothesizing a normal distribution:
  (6) $$b_{j}=\sum_{k=1}^{K}w_{jk}\;\text{N}\left(\mu_{jk},\Sigma_{jk}\right)$$
  where *w_jk_* is, for each state *j*, amixture coefficient, weighting *K* multivariate normal distributions N, with mean *μ_jk_* and covariance matrix expressed by Σ*_MxM_* = {Σ*_jk_*}.
- **π** is the initial state vector distribution:
  (7) $$\begin{array}{cc} \pi ={\left\{\pi_{i}\right\}}_{Qx1} & \pi_{i}=P\left(X\left(t_{0}\right)=S_{i}\right) \end{array}$$
  The use of a cHMM as a feature classifier requires the two following phases. The first phase consists in the collection of a training data-set for the computation of the model parameters **λ**. The Baum-Welch algorithm [36], which is popular for tackling this problem, is used in the present paper.
  The second phase, based on the results obtained during the first phase, allows featureclassification. The Viterbi algorithm is the best candidate for the classification and we adopted a“forward-only” modification of this method to obtain real-time processing[5]. The“forward-only” is applied to each signal in order to find the *l*-th state of likely sequence *l_t_n__* and the probability associated at each *i*-th state *γ_t_n__* (*i*); in particular the algorithm is composed of three steps, as following:
- Initialization
  (8) $$\begin{array}{cc} \gamma_{t_{0}}\left(i\right)=\pi_{i}b_{i}\left(\text{Y}\left(t_{0}\right)\right) & 1\leq i\leq N\\ l_{t_{0}}=\arg\max\left[\gamma_{t_{0}}\left(i\right)\right] & \end{array}$$
- Recursion
  (9) $$\gamma_{t_{n}}\left(i\right)=\max\left[\gamma_{t_{n}-1}\left(i\right)a_{ij}\right]b_{i}\left(Y\left(t_{n}\right)\right)$$
- Likely sequence
  (10) $$l_{t_{n}}=\arg\max\left[\gamma_{t_{n}}\left(i\right)\right]$$

#### 2.1.2. Scalar (SC) and Vectorial Classifiers (VC)

Gait cycle is generally divided in four gait phases, Flat Foot, Heel Off, Swing, and Heel Strike, which represent the hidden states of here adopted cHMM [5,16]; in normal gait, walking phases occur following the above-reported sequence. State transitions follow a left-right model and, consequently, transition matrix **A**, implemented in the present study, is [5]:

(11) $$\text{A}=\left\{a_{ij}\right\}=\left[\begin{array}{cccc} 0.9 & 0.1 & 0 & 0\\ 0 & 0.9 & 0.1 & 0\\ 0 & 0 & 0.9 & 0.1\\ 0.1 & 0 & 0 & 0.9 \end{array}\right]$$

**Figure 1.**
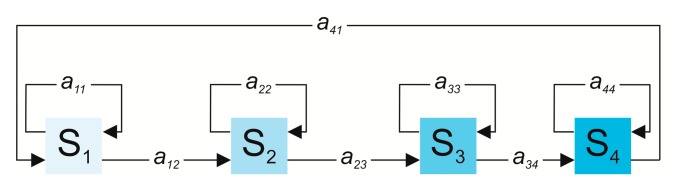
Possible transitions (a_ij_) among four states of cHMM (S_i_) according to a left-right model.

Since transitions are very quick with respect to the gait cycle, they are less frequent in the current state sequence; thus, diagonal elements assume higher values than the others [13] and in Figure 1 the possible transitions among gait phases are reported.

Because the initial state of the model at time *t_0_* is not known, its distribution can be chosen as:

(12) $$\pi \left(t_{0}\right)=\left[\begin{array}{c} 0.25\\ 0.25\\ 0.25\\ 0.25 \end{array}\right]$$

This means that each state has the same probability of being the first in a state sequence.

The VC is based on a multivariate Normal distribution:

(13) $$p_{t_{n}}\left(\text{Y}\left(t_{n}\right)|\mu \left(t_{n}\right),\Sigma \left(t_{n}\right)\right)=\frac{1}{{\left(2\pi \right)}^{\overset{M}{\;}/\underset{2}{\;}}{\left|\Sigma \left(t_{n}\right)\right|}^{\overset{1}{\;}/\underset{2}{\;}}}e^{\left\{-\frac{1}{2}{\left(\text{Y}\left(t_{n}\right)-\mu \left(t_{n}\right)\right)}^{T}\Sigma {\left(t_{n}\right)}^{-1}\left(\text{Y}\left(t_{n}\right)-\mu \left(t_{n}\right)\right)\right\}}$$

where at a given time *t_n_*:

- **Y**(*t_n_*) is the observation value [1 × M];
- **μ**(*t_n_*) is the vector of mean values [1 × M];
- **Σ**(*t_n_*) is the covariance matrix [M × M].

When cHMM is applied to one signal (M = 1), the VC is coincident with the SC. SC is based on a univariate Normal distribution and the Equation (13) can be rewritten as:

(14) $$p_{t_{n}}\left(Y\left(t_{n}\right)|\mu \left(t_{n}\right),\sigma \left(t_{n}\right)\right)=\frac{1}{\sigma \left(t_{n}\right)\sqrt{\left(2\pi \right)}}e^{\left\{\frac{-{\left(Y\left(t_{n}\right)-\mu \left(t_{n}\right)\right)}^{2}}{2\sigma {\left(t_{n}\right)}^{2}}\right\}}$$

where at a given time *t_n_*, *σ*(*t_n_*) is the standard deviation.

By applying the “forward-only” algorithm proposed by Abaid *etal.* [5] to SC andVC, two parameters are calculated: (i) the most likely sequence of states (**L***^SC^* and **L***^VC^*), composed of each likely state *l*(*t_n_*) at time *t_n_*; (ii) the probability associated at each *l*-th state ( $\gamma_{l}^{SC}\left(t_{n}\right)$ and $\gamma_{l}^{VC}\left(t_{n}\right)$).

#### 2.1.3. Distributed Classifier (DC)

**Figure 2.**
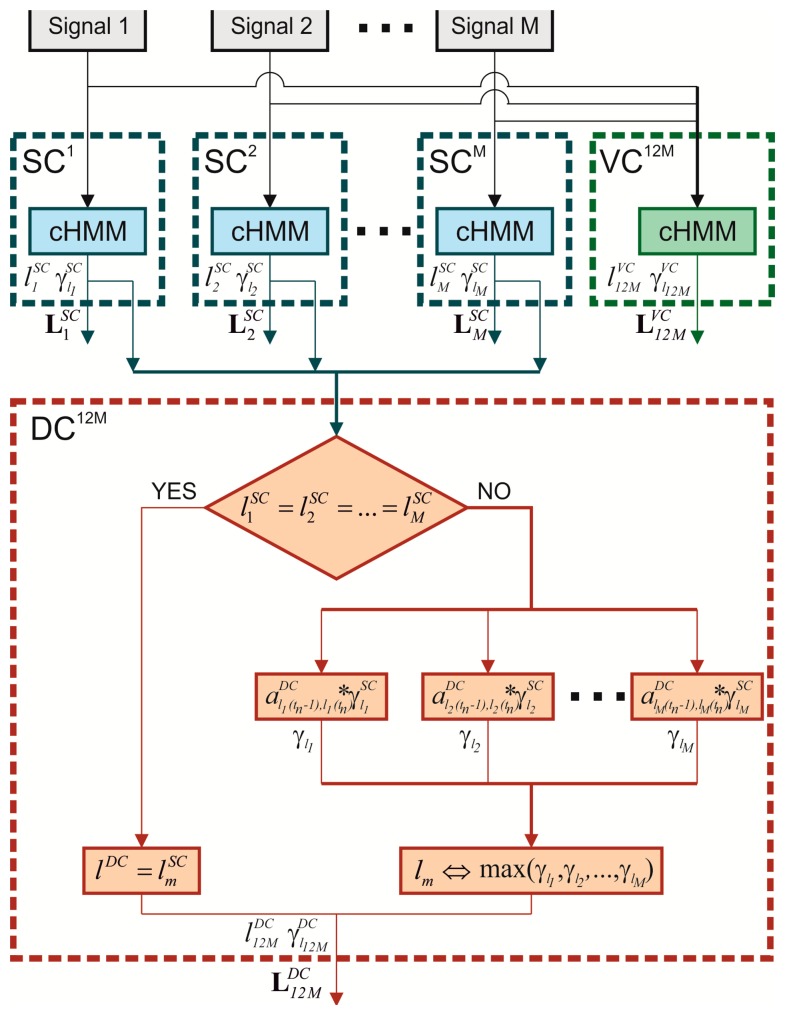
Logical diagram of Scalar Classifier SC, Vectorial Classifier VC and Distributed Classifier DC.

The novel contribution of the present study is the implementation of a new algorithm to detect gait phases based on a distributed stochastic model and on a hierarchical-weighted classification. The algorithm, as shown in Figure 2, is based on the processing of each **L***^SC^* simultaneously obtained from more than one sensor in order to obtain the final likely sequence of states **L***^DC^*.

Taking into account a number of signals equal to M, for each time*t_n_* and each signal *m*, the DC compares each state $l_{m}^{SC}\left(t_{n}\right)$ following the above if-structure:

If

(15) $$l_{1}^{SC}\left(t_{n}\right)=l_{2}^{SC}\left(t_{n}\right)=\ldots =l_{M}^{SC}\left(t_{n}\right)$$

then

(16) $$l^{DC}\left(t_{n}\right)=l_{m}^{SC}\left(t_{n}\right)$$

Conversely, if at least a disagreement is found at *t_n_*, for each *m* signal, a new probability $\gamma_{l_{m}}^{SC}\left(t_{n}\right)$ is calculated by the introduction of a distributed transition matrix ${\text{A}}^{DC}=\left\{a_{ij}^{DC}\right\}$:

(17) $$\gamma_{l_{m}}\left(t_{n}\right)=a_{l_{m}\left(t_{n-1}\right),l_{m}\left(t_{n}\right)}^{DC}*\gamma_{l_{m}}^{SC}\left(t_{n}\right)$$

Then, *l^DC^*(*t_n_*) is equal to $l_{m}^{SC}\left(t_{n}\right)$ which is obtained by the *m*-th signal characterized by the maximumvalue of *γ_l_m__* (*t_n_*).

By means of the repetition of the if-structure for each *t*, the final likely sequence of states **L***^DC^* can be individuated. The performance of the algorithm is strictly related to the choice of the distributed transition matrix **A***^DC^* implemented in the distributed classifier.

### 2.2. Experimental Procedure

Ten healthy subjects (26.2 ± 23.1 years), who had no known gait or other pathologies influencing their walking patterns, were enrolled in this study at the Movement Analysis and Robotics Laboratory (MARlab) of the Bambino Gesù Children's Hospital. Informed consent, in written form, was obtained from the participants. The Research Ethics and Medical Board of the Bambino Gesù Children's Hospital approved the experimental protocol. The protocol conforms to the ethical standards outlined in the 1964 Declaration of Helsinki.

**Figure 3.**
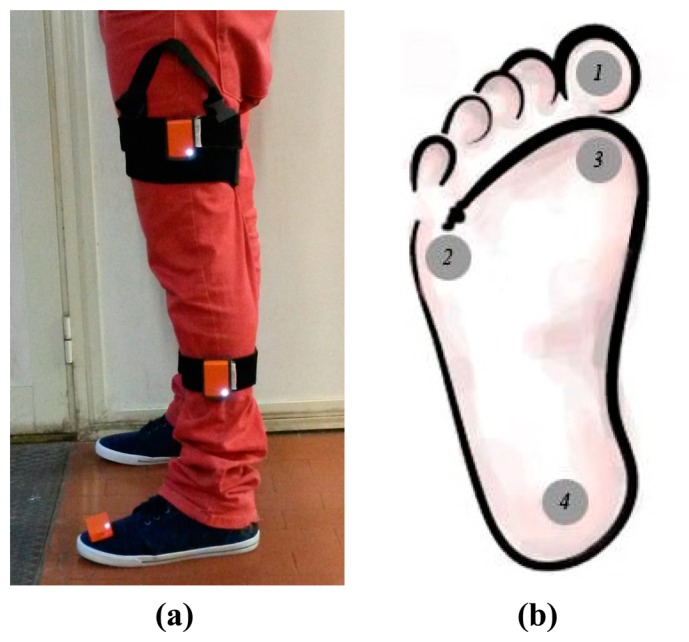
(**a**) Position of IMUs on participants' left lower limb; (**b**) position of foot switches (1: toe, 2: fifth metatarsophalangeal, 3: first metatarsophalangeal, and 4: heel).

The participants' left lower limb was equipped with three Inertial Measurement Units (XBus Master, Xsens Technologies, The Netherland) on foot, shank, and thigh, as reported in Figure 3, and four footswitch sensors (Footswitch FSR sensors, Wave Cometa, Italy) on the foot sole, at the toe, at the heel and at the first and fifth metatarsophalangeal articulations, as reported in Figure 3b. As regards the IMU system, only the outputs of gyroscopes were used for the post-processing.

Since only gyroscope data were acquired from IMU sensors, no particular procedure was needed for Kalman filter stabilization in order to limit the effects induced by indoor magnetic distortions [39]. An operator precisely positioned the sensors on the subject, by aligning one of their sensible axes with the sagittal axis of each body segment. This alignment methods is easier to be performed than a functional calibration procedure [40–42] and it gives better results when only one axis for each sensor has to be taken into account. Thus, we decided to use the manual alignment because we analyzed only the sagittal component of angular velocity.

Sagittal angular velocities of the three body segments were captured through the tri-axial gyroscope into each IMU at a frequency of 60 Hz, while the actual sequence of gait phases was captured by means of foot switches at a frequency of 2 kHz.

Participants were asked to perform a walking task on a treadmill for at least 120 s at their preferred speed, in the range of 0.5–1.5 m/s; this range was chosen to guarantee a normal pace and prevent fatigue [43]. Each subject self-selected the preferred velocity by walking on the treadmill during a preliminary session before the walking task. The mean value of the chosen treadmill speed was 0.81 ± 0.08 m/s for all the subjects involved in the study. The actual data acquisition began five seconds after reaching the speed chosen by the subject and ended five seconds before the turning off of the treadmill to prevent acquisition during the transient state. The task was repeated three times. Between trials, the subject remained on the treadmill in standing position. The entire experiment, including instrumentation, walking tasks, and de-instrumentation, was completed within one hour by all participants. All participants completed the tasks without expressing fatigue.

### 2.3. Data Processing

**Figure 4.**
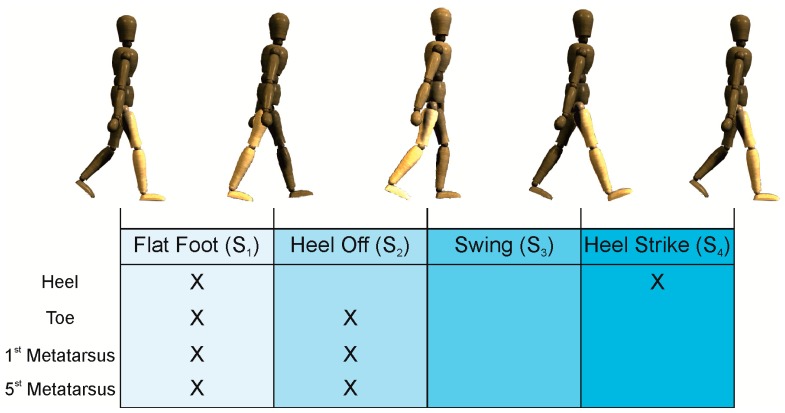
Combination of active foot switches, marked with X, to detect gait phases.

Data processing and data analysis were performed using MATLAB software (MathWorks, Natick, MA, USA) and a Sony Vaio with Windows 7 Home Premium 64 bit (IntelCore i5 2410 M, CPU@2.30 Ghz, Minato, TKY, Japan). Each gait cycle was subdivided into four phases, which were used as the states of cHMM: Flat Foot, Heel Off, Swing, and Heel Strike. In particular, Flat Foot corresponds to the state where the foot sole is in contact with the floor; during the Heel Off phase the heel is not in contact with the floor; Swing occurs when the foot is in the air; Heel Strike corresponds to the state where only the heel is in contact with the floor. Each phase has been recognized by means of the identification of which foot switches were activated such as reported in Figure 4.

**Figure 5.**
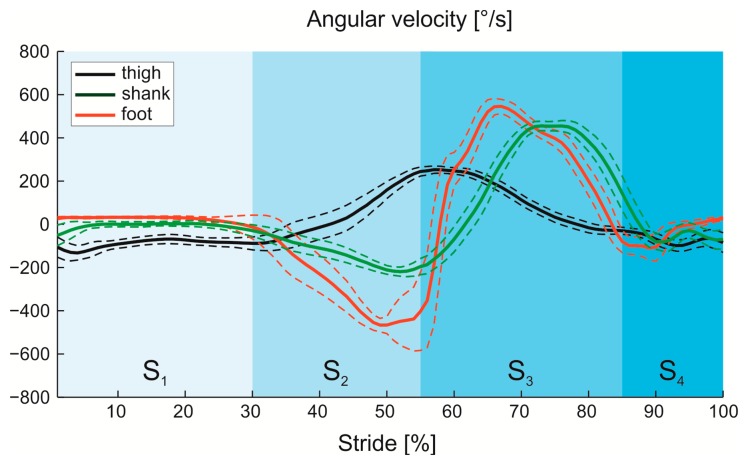
**Participant #1:** Mean and standard deviation of angular velocity of foot (red), shank (green) and thigh (black) partitioned into the four gait phases (S_1_: Flat Foot, S_2_: Heel Off, S_3_: Swing, S_4_: Hell Strike).

The output of the gyroscopes, which represents the observation **Y** of cHMM, was, firstly, treated with a low-pass Butterworth filter with 15 Hz cut-off frequency, and then partitioned into the four gait phases by means of the foot switch data [5]. After the normalization of the time length of each phase, mean and standard deviation of the angular velocities were calculated and used to train parameters of cHMM, according to the Baum-Welch algorithm. In Figure 5 the paradigmatic angular velocities of foot, shank and thigh are reported for one subject.

For each subject, a leave-one-out cross-validation analysis was applied to the three walking trials to validate cHMM in a recursive manner. Specifically, the model was trained by two trials, and the remaining one was used as the validation data. The procedure was repeated for all trials in turn [16]. After the data validation procedure, we obtained the likely state sequences **L***^SC^*, **L***^VC^*, and **L***^DC^* for the three here examined classifiers. We chose a leave-one-out cross-validation because it is a model validation technique frequently used to estimate the generalization and capabilities of a classifier [44].

### 2.4. Data Analysis

The choice of the number of sensors and the body segments where the sensors were placed-foot (f), shank (s), and thigh (t)-allowed us to compare the performance of 11 classifiers: a cluster of three Scalar Classifiers (SC^f^, SC^s^, and SC^t^); a cluster of four Vectorial Classifiers (VC^fs^, VC^ft^, VC^st^, and VC^fst^); and finally, a cluster of four Distributed Classifiers (DC^fs^, DC^ft^, DC^st^, and DC^fst^).

We computed True Positive Rate (*TPR*) and True Negative Rate (*TNR*) relative to each of the three classifiers here examined, assuming the foot switch signals as a reference [5,16]. *TPR* and *TNR* represent the sensitivity and specificity of the classifier in the detection of gait phases. In particular, the phase transitions that are similarly detected by classifier and reference signal were considered as a True Positive, otherwise they were considered as a False Positive. The non-transitions that are similarly detected by classifier and reference signal were considered as a True Negative, otherwise as a False Negative. *TPR* and *TNR* are defined as:

(18) $$\text{TPR}=\frac{\text{True Positive}}{\text{True Positive}+\text{False Negative}}$$

(19) $$\text{TNR}=\frac{\text{True Negative}}{\text{False Positive}+\text{True Negative}}$$

Both indices were calculated using a tolerance window of 60 ms centered at each time step [5,45].

To estimate the weight of each sensor in the hierarchical classification of Distributed Classifier DC, a Decision Index was calculated. It represents how many times each sensor took the final decision in DC and it was expressed as a percentage of the total number of possible decisions. Decision Index was calculated for the four combinations of DC and for all subjects, then mean and standard deviation values were calculated.

To estimate the time processing required for each classifier in order to recognize the gait phases, the Computational Load was calculated as the time spent by each classifier to estimate a state S_i_ of the state sequence. Computational Load was calculated for all the classifiers and for all subjects, consequently mean and standard deviation values were calculated.

To estimate the performance of DCs to evaluate the gait variability, we calculated Mean Time (*MT*) and Coefficient of Variation (*CoV*) of stride and of each gait phase. For the computation of the previous two indices, firstly the sequences of state, which are the outputs of DCs, were partitioned into the corresponding gait phases, then mean (*MT*) and standard deviation (std) of stride time and of each phase duration were evaluated for each walking task and for each subject. *CoV* [46] is defined as:

(20) $$\text{CoV}=\frac{\text{std}}{MT}\times 100\left[\%\right]$$

*MT* and *CoV* were also calculated for the reference signal (FSR) in order to compare them with the ones obtained from DCs.

### 2.5. Statistical Analysis

All data were tested for normality with the Shapiro-Wilk test. One-way ANOVA tests were conducted in order to find noteworthy differences among the 11 classifiers both for *TPR* and *TNR*. Differences among FSR and DCs in the evaluation of *MT* and *CoV* were also analyzed with one-way ANOVA tests. Statistical difference was set at 0.05. When significant differences were found, a Bonferroni's test for multiple comparisons was performed. Furthermore, to evaluate reliability Intra-class Correlation Coefficients (ICC) for *MT* and *CoV* were calculated. In accordance with literature [47], values in the range 0.0–0.4 were considered poor, 0.40–0.59 fair, 0.60–0.74 good, and 0.75–1.00 to be excellent.

The software package SPSS (IBM-SPSS Inc., Armonk, NY, USA) was used.

### 2.6. Preliminary Study for the Choice of the Matrix **A**^DC^

The selection of the distributed transition matrix **A***^DC^*(Equation (17)) is crucial for the validation of the novel distributed classifier. The best **A***^DC^* was chosen testing two different matrices. Firstly, we implemented a left-right model [36], where ${\text{A}}_{LR}^{DC}$ can be written as:

(21) $${\text{A}}_{LR}^{DC}=\left[\begin{array}{cccc} p & 1-p & 0 & 0\\ 0 & p & 1-p & 0\\ 0 & 0 & p & 1-p\\ 1-p & 0 & 0 & p \end{array}\right]$$

Secondly, we tested a matrix ${\text{A}}_{\text{RLR}}^{DC}$ characterized by the possibility of passing from the current state to the previousone. This matrix, defined as Right-Left-Right, could be useful in the case where, due to a not negligible sensor noise, the actual state is misclassified and addressed as the following one. At the successive step of the algorithm, a simple left-right matrix ${\text{A}}_{LR}^{DC}$ would not allow going backwards, and, consequently, it determines a misclassification of the algorithm until the same transition phase in the next gait cycle occurs. A Right-Left-Right model can be written as:

(22) $${\text{A}}_{\text{RLR}}^{DC}=\left[\begin{array}{cccc} p & \frac{1-p}{2} & 0 & \frac{1-p}{2}\\ \frac{1-p}{2} & p & \frac{1-p}{2} & 0\\ 0 & \frac{1-p}{2} & p & \frac{1-p}{2}\\ \frac{1-p}{2} & 0 & \frac{1-p}{2} & p \end{array}\right]$$

**Figure 6.**
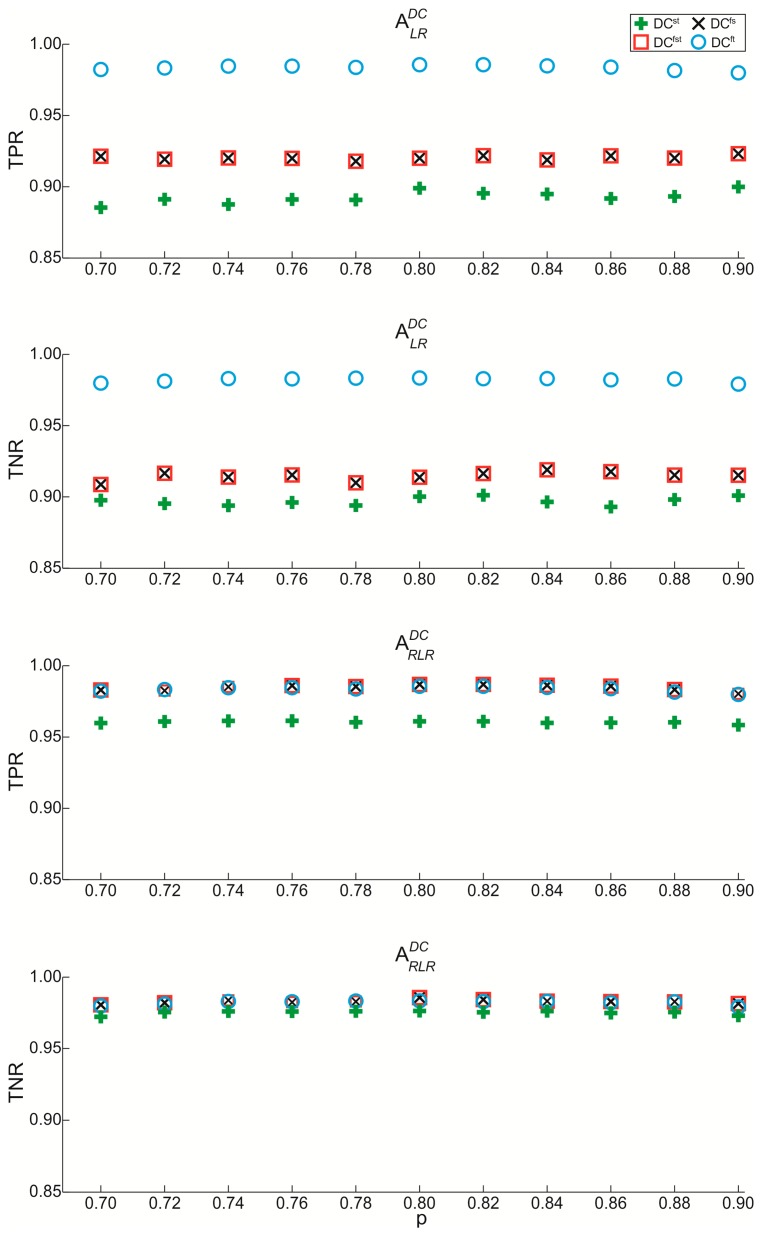
Values of True Positive Rate (*TPR*) and True Negative Rate (*TNR*) evaluated for ${\text{A}}_{LR}^{DC}$ and ${\text{A}}_{\text{RLR}}^{DC}$ as a function of *p* values for the four combinations of DC.

Values of probability *p* between 0.07 and 0.09 in steps of 0.02 were tested ondata acquired from all subjects for the two above reported matrices. *TPR* and *TNR* were evaluated both for ${\text{A}}_{LR}^{DC}$ and for ${\text{A}}_{\text{RLR}}^{DC}$ to find the best distributed matrix that can be implemented in the distributedclassifier. In Figure 6, *TPR* and *TNR* values evaluated for matrices ${\text{A}}_{LR}^{DC}$ and ${\text{A}}_{\text{RLR}}^{DC}$ are represented.

*TPR* and *TNR* assume similar values in the range of tested*p* both for ${\text{A}}_{LR}^{DC}$ and ${\text{A}}_{\text{RLR}}^{DC}$. Performing the comparison between matrices implemented in DC^st^,DC^fs^, and DC^fst^, *TPR*, and *TNR* for ${\text{A}}_{LR}^{DC}$ reach lower values than one evaluated by means of ${\text{A}}_{\text{RLR}}^{DC}$ of about 6%. The observed differences are caused by the noise, whichimplies a misclassification of DCs. The matrix ${\text{A}}_{LR}^{DC}$ can be implemented in place of ${\text{A}}_{\text{RLR}}^{DC}$ only when the sensors placed on foot and thigh are used (DC^ft^). Therefore, for the application of experimental protocol, any value of *p* included between 0.7 and 0.9 can be utilized; in particular we chose, from Equation (22), the following matrix:

(23) $${\text{A}}_{\text{RLR}}^{DC}=\left[\begin{array}{cccc} 0.8 & 0.1 & 0 & 0.1\\ 0.1 & 0.8 & 0.1 & 0\\ 0 & 0.1 & 0.8 & 0.1\\ 0.1 & 0 & 0.1 & 0.8 \end{array}\right]$$

## 3. Results

In Table 1 mean and standard deviation values of *TPR* and *TNR*, as well as statistical differences (p < 0.05) are reported for all tested combinations of the three classifiers here examined.

With regards to the Scalar Classifiers, statistical differences among all SCs were found; in particular, *TPR_SC^f^_* and *TPR_SC^f^_* showed the highest values, while *TPR_SC^t^_* and *TPR_SC^t^_* showed the lowest. Regarding the Vectorial Classifiers, although *TPR_VC^st^_* and *TNR_VC^st^_* values were high, *i.e.*, 0.97 and 0.96 respectively, they were statistically lower than the ones measured with the other Vectorial Classifiers. Moreover, *TPR_VC^fst^_* and *TNR_VC^fst^_* showed maximum values, but they were not statistically different from ones evaluated with VC^fs^ and VC^ft^. Taking into account the Distributed Classifiers, *TPR_DC^st^_* and *TNR_DC^st^_* were statistically lower than the same parameters evaluated with the other DC. Moreover, DC^fs^ and DC^fst^ showed higher values of specificity and sensitivity that were not statistically different from the ones evaluated with DC^ft^. Analyzing the three classifiers together, both parameters evaluated with SC^s^ and SC^t^ were statistically different from ones obtained with all other combinations of VC and DC. On the contrary, *TPR_SC^f^_* and *TPR_SC^f^_* did not show any differences comparing them with specificity and sensitivity measured with all combinations of VC and DC. Moreover, for each combination of sensors, *TPR_VC_* and *TPR_VC_* were not statistically different from *TPR_DC_* and *TPR_DC_*.

Table 2 shows Decision Index values calculated for all Distributed Classifiers. The value of Decision Index measured for the thigh is 0.00% for DC^ft^ and DC^fst^ and this means that the sensor placed on the thigh did not take part in the decision-making process. The sensor on the foot, in all combinations in which it was present, made the decision in most cases. In the absence of the sensor placed on foot, *i.e.*, DC^st^, the final decision was made mainly by the sensor placed on the shank.

In Table 3 values of Computational Load are reported. The results showed that all DCs did not present any difference with respect to Computational Load values evaluated with all SCs; moreover, VCs showed the highest values of Computational Load that appeared to be on the order of about 10^2^ larger than the others.

**Figure 7.**
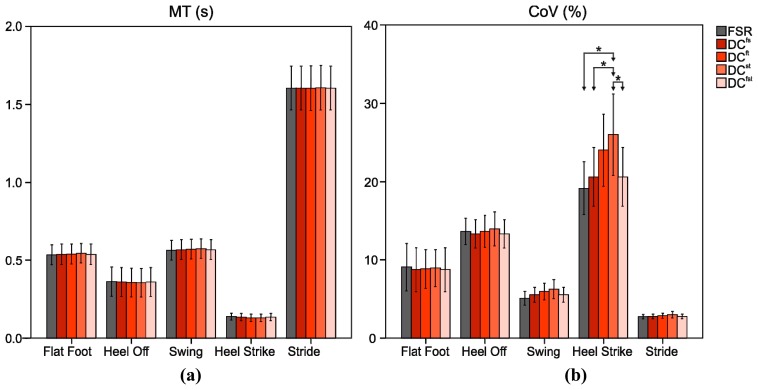
(**a**) Mean Time (*MT*) and (**b**) Coefficient of Variation (*CoV*) evaluated for stride and each gait phase for FSR and DCs. “*” indicates statistically significant differences.

In Figure 7 mean and standard deviation of *MT* and *CoV* for stride and each gait phase as well as statistical differences (p < 0.05) are reported for FSR and DCs. As regards *MT* no statistically significant differences were observed among DCs and FSR. DCs and FSR detected a Stride *MT* equal to 1.60 ± 0.13 s; in particular *MT* assumes the lowest values for Heel Strike and the highest ones for the Flat Foot/Swing. Taking into account *CoV*, significant differences were found in the evaluation of Heel Strike *CoV* between DC^st^ and FSR, DC^fs^, and DC^fst^. In the Stride and in the other gait phases no statistically significant differences were found among FSR and DCs. In particular, Heel Strike *CoV* reached higher values (always higher than 19%), while Stride *CoV* assumed lower values (always less than 3%).

In Table 4, values of ICC for *MT* and *CoV* are reported. The results showed that ICC for *MT* was always more than 0.97, while ICC for *CoV* reached lower values, but always in the range of good or excellent reliability.

## 4. Discussions

### 4.1. Scalar Classifier

SC shows the best performance when the sensor has been placed on the foot (SC^f^). This reflects the conclusion of Abaid et colleagues [5], in which authors stated that the classifier based on the angular velocity of the foot is sufficient for the identification of gait phases in comparison with the other scalar classifiers. In reality, SC^s^ could be also used, even though this would lead to a decrease in sensitivity and specificity performance of about 4%, which limits use when the highest accuracy is required. In addition, SC^t^ achieves performance significantly lower than others, and in particular small value of specificity causes an increase of the detection of false transitions; it implies a shorter duration for each gait phase with an increase in the number of continuous transitions among states.

When the classifier is implemented to control gait phases in active orthoses, as also reported by Abaid *et al.* [5], the detection of false transition could determine an incorrect actuator control, causing lack of synchrony between the patient's needs and the assistance offered by the device. In fact, active orthoses for ankle rehabilitation are designed to assist impaired gait by providing assistance at the beginning of propulsion phase, *i.e.*, the end of Flat Foot, and by supporting the foot in the middle and at the end of swing phase to prevent the drop-foot [48–50]. This means that low values of specificity would provide wrong information on the actual gait phases and, consequently, a not-suitable assistance to patients. The significant differences among the three scalar classifiers is due to the variability of angular velocity of foot, shank and thigh. As reported in Figure 5, the angular velocity of each body segment shows different variability related to each gait phase. From an examination of the figure, it emerges that the angular velocity of the foot presents, unlike the others, the greatest differences between gyroscope signal patterns in the four phases, as also reported in [16]. In order to detect gait phases, it is sufficient to train cHMM only once with the subject instrumented with FSRsfoot switches, and then the classifier based on gyro signals of the foot is able to detect gait phases in post-processing without any kind of operator intervention. Conversely, training process is not sufficient for gyros applied on the shank and the thigh, which cannot be used as classifiers for gait detection, due to high possibility of detecting false transitions.

In conclusion, SC^f^ is the only combination among SCs usable as classifier to detect gait phases with acceptable performance.

### 4.2. Vectorial Classifier

As regards the vectorial classifiers, the highest values of *TPR* and *TNR* are reached using all sensors placed on the lower limb (VC^fst^). Moreover, these values are comparable with the sensitivity and specificity obtained with VCs based on two sensors where one is placed on the foot, *i.e.*, VC^fs^ and VC^ft^. It implies that, when a gyroscope is mounted on the foot, the increase in performance due to the utilization of the third sensor can be considered negligible. Despite the fact that the angular velocities of shank and thigh are less variable during gait, VC^st^ provides values higher than 0.96 for both parameters. This implies that, in the absence of the contribution of a sensor placed on the foot, the two angular velocities can train the parameters of cHMM to recognize gait phases, providing performance comparable to those obtained with the other VCs.

In conclusion, all of VCs can be considered as classifiers when gait recognition is required.

### 4.3. Distributed Classifier

Comparing the performance of all DCs, it appears that DC^st^ is the worst classifier in terms of sensitivity and specificity although its values are always higher than 0.95%. This implies that the lack of information relating to the angular velocity of foot causes a decrease in performance, but the values of *TPR* and *TNR* remain acceptable for robust gait detection. In addition, the performance of DC^fs^ are the same as the ones obtained with DC^fst^; this implies that the sensor place on the thigh never took the decision in the DC^fst^ algorithm. It is also confirmed by the Decision Index of foot and shank, which assume the same values for DC^fs^ and DC^fst^ and the Decision Index of thigh that is always equal to zero in DC^fst^. Actually, the Decision Index reflects the level of variability of the measured angular velocities. In fact, the probability associated at the states estimated with angular velocity of foot is always higher than the one estimated with other sensors. The sensor on the thigh takes part in decisions only when the sensor on the foot is not connected. As also reported for VC^st^, the small variability of angular velocity of the thigh makes its decision possible only in DC^st^.

In conclusion, all DCs can be taken into account as classifiers for gait detection.

### 4.4. Comparison between SC, VC, and DC

As regards the comparison between the SCs and VCs, the statistical differences among parameters evaluated with VC^st^, SC^s^ and SC^t^, imply that, in the absence of the sensor placed on the foot, the use of a VC is mandatory in order to reach the highest values of *TPR* and *TNR*. However, when the algorithms involve the use of the angular velocity of the foot, VCs do not significantly improve the capability of detecting gait phases compared with SC^f^; this result allows the utilization of a single sensor with respect to more complex configurations of sensors avoiding a further increase of computational complexity.

Comparing DCs and SCs, statistical differences were found among each DC and both SC^s^ and SC^t^. This implies that, as also reported in [28], the use of a hierarchical classification based on a data fusion procedure causes an improvement in performance with respect to the most parts of ones based on the analysis of a single signal. In particular, the main peculiarity of the use of a distributed classifier with respect to scalar ones is pinpointed when the performance of DC^st^ are compared with those of both SC^s^ and SC^t^; actually, when the foot is not sensorized, the fusion of the individual characteristics of scalar signals determines a significant increase of both *TPR* and *TNR*. This finding confirms that DC can be used as classifier for gait detection when the signal of foot angular velocity is not available. Moreover, in the presence of a sensor placed on the foot, DCs show the same behavior as VCs when they are compared with SCs. This implies that DCs can be considered useless for the detection of gait phases when almost a gyroscope is placed on foot. Examining DC^ft^ and SC^f^, it emerges that the two classifiers show the same values of specificity and sensitivity. This can be explained by analyzing the values of Decision Index for DC^ft^, which is equal to zero for the sensor placed on the thigh. Therefore, only the sensor on the foot takes the final decision in the hierarchical algorithm based on thigh and foot sensors with the consequently coincident performances of SC^f^ and DC^ft^.

Comparing the performance of VCs and DCs in terms of specificity and sensitivity, no significant difference was found between each combination of sensors. It confirms the quality of the novel distributed classifier that shows performance comparable with the vectorial one. However, taking into account the Computational Load parameter, it emerges that all of VCs are characterized by the highest values with respect to those of DC. Therefore, VCs show an increase of processing time mainly due to the evaluation of the multivariate probability distribution, which is computationally more demanding, during each step of cHMM. Thus, the high value of computational time determines the limitation of the use of VCs only in an off-line recognition of gait phases. Conversely, when a real-time detection of gait is required, only DCs can be considered as powerful classifiers since they show values of Computational Load of about 9 ms that are comparable with the ones obtainable with SCs. It is important to note that our findings related to Computational Load are dependent on the type of the chosen processor and software.

DCs show performance comparable with VCs in terms of sensitivity and specificity, and computational loads equal to the ones calculated with SCs. The high values of sensitivity and specificity and the low value of processing time obtained with DCs can permit its implementation in the real-time control of active orthoses of knee, such as [51,52], in order to block the rotation of the joint during foot flat phase and to allow free motion during the swing phase [53]. Consequently, the implementation of the DC allows us to avoid the use of additional sensors mounted on the foot, simplifying the design of active orthoses for the recovery of knee mobility. By contrast, in case of active orthoses of the ankle, the cHMM applied to the angular velocities of the foot can be considered sufficient to correctly detect gait phases.

In conclusion, only the novel hierarchical algorithm herewith proposed can be useful to control the movement of active orthoses of the knee if the robotic devices are sensorized at the shank and thigh and not at the foot.

### 4.5. Are DCs Reliable in Study of Gait Variability?

The analysis of *MT* values related to Flat Foot, Heel Off, Swing and Heel Strike confirmed the results reported by Abaid *et al.* [5] as regards the percentage of time spent by each phase during the stride; in particular Heel Strike has the less duration, while Swing and Flat Foot are characterized by the highest time length. In the evaluation of *MT*, we found no statistically significant differences between all combinations of DC and FSR, this implies that *MT* values are comparable between DCs and FSR, agreeing to within a few percent. Moreover, the analysis of ICC confirms the excellent reliability of the novel distributed classifier in the evaluation of the gait variability based on *MT*.

As regards the analysis of *CoV*, the Stride *CoV* was never higher than 3%, confirming values reported in literature, both for adults [54] and for children over 11 years old [46]. Furthermore *CoV* values for Stride and all gait phases decreased when the time length increased; it is in accordance to the findings of Liu *et al.* [55] where higher values of *CoV* were measured in gait phases of less duration. Differently from *MT*, we found statistically significant differences between DC^st^ and FSR, DS^fs^ and DC^fst^ in the evaluation of Heel Strike *CoV*, while no differences were found between FSR and DCs in the other gait phases and in Stride. This outcome implies that in studies of gait variability, that required the analysis of *CoV*, DCs are comparable to FSR, except for the evaluation of Heel Strike *CoV*, in which DC^st^ is not able to provide the same performance of other classifiers. This finding confirms that DC^st^ is the worst classifier among DCs as also emerged from the outputs of the ANOVA tests applied to *TPR* and *TNR*. Even if we demonstrated that, in most cases, DCs are comparable to FSR, the error in the estimation of the likely state sequence of the novel distributed classifier could compromise the studies of gait variability based on *CoV*. In fact, the classification of false transitions or false non-transitions implies the identification of gait phases shorter or longer than the actual duration, with an increase of standard deviations and, consequently, of the *CoV* values. As regards the ICC analysis of *CoV*, no differences were found between the novel distributed classifiers and the reference measurement system even if the ICC values appeared to be lower than the ones evaluated for *MT*. Nevertheless, ICC values were in the range of good or excellent in all cases. This findings confirmed the lower reliability of *CoV* evaluation with respect to others time-variability parameters [56].

In conclusion, considering the observed performance of DCs with reference to FSR, the novel algorithm could also represent a useful tool in identifying and quantifying gait variability also in a clinical perspective to select a patient specific treatment.

## 5. Conclusions

This study presents a novel gait detection algorithm based on hierarchical weighted decision, addressed as a Distributed Classifier, applied to process data generated by uni-axial gyroscopes. The innovation of the Distributed Classifier algorithm is on the implementation of a data‐fusion procedure using the angular velocity signals of two or three gyroscopes placed on the lower limbs. Our findings show that the here proposed and validated Distributed Classifier applied to each combination of gyroscopes can successfully detect gait phases with better performance when each DC has been compared with a Scalar Classifier of both shank and thigh. Furthermore, the performance of each DC can be considered similar to the ones obtainable with a Vectorial Classifier applied to the same signals; regardless VCs, DCs can, instead, also be implemented in the real-time application of gait phase recognition, such as the control of active orthoses for the recovery of the mobility of lower limb joints and in the quantification of gait variability.

## Figures and Tables

**Table 1.** Means, standard deviations of True Positive Rate (*TPR*) and True Negative Rate (*TNR*), and statistical differences for all combinations of Scalar Classifier (SC), Vectorial Classifier (VC), and Distributed Classifier (DC). “All” indicates statistically significant differences between the specific algorithm and the others.

**Algorithm**	**TPR**		**TNR**	
	
	**Mean (std)**	**Differences**	**Mean (std)**	**Differences**
SC^f^	0.98 (0.01)	SC^s^, SC^t^	0.98 (0.02)	SC^s^, SC^t^
SC^s^	0.94 (0.01)	All	0.93 (0.02)	All
SC^t^	0.83 (0.01)	All	0.77 (0.01)	All

VC^fs^	0.99 (0.01)	SC^s^, SC^t^, VC^st^, DC^st^	0.98 (0.01)	SC^s^, SC^t^, VC^st^, DC^st^
VC^ft^	0.98 (0.01)	SC^s^, SC^t^	0.98 (0.02)	SC^s^, SC^t^
VC^st^	0.97 (0.01)	SC^s^, SC^t^, VC^fs^, VC^ft^, VC^fst^	0.96 (0.02)	SC^s^, SC^t^, VC^fs^, VC^ft^, VC^fst^
VC^fst^	0.99 (0.01)	SC^s^, SC^t^, VC^st^, DC^st^	0.99 (0.01)	SC^s^, SC^t^, VC^st^, DC^st^

DC^fs^	0.99 (0.01)	SC^s^, SC^t^, DC^st^	0.98 (0.01)	SC^s^, SC^t^, DC^st^
DC^ft^	0.98 (0.01)	SC^s^, SC^t^	0.98 (0.02)	SC^s^, SC^t^
DC^st^	0.96 (0.02)	SC^s^, SC^t^, VC^fs^, VC^fst^, DC^fst^, DC^fs^	0.95 (0.02)	SC^s^, SC^t^, VC^fs^, VC^fst^, DC^fst^, DC^fs^
DC^fst^	0.99 (0.01)	SC^s^, SC^t^, DC^st^	0.98 (0.01)	SC^s^, SC^t^, DC^st^

**Table 2.** Means, standard deviations of Decision Index for all of combinations of Distributed Classifier (DC).

**Body Segment**	**Decision Index (%)**			

	**DC^fs^**	**DC^ft^**	**DC^st^**	**DC^fst^**
Foot	85.45 (2.35)	100.00 (0.00)	/	85.45 (2.35)
Shank	14.55 (2.35)	/	76.97 (1.55)	14.55 (2.50)
Thigh	/	0.00 (0.00)	23.03 (1.55)	0.00 (0.00)

**Table 3.** Means of Computational Load.

**Classifier**	**Computational Load (s)**
SC^f^, SC^s^, SC^t^	0.009
VC^fs^, VC^ft^, VC^st^	0.147
VC^fst^	0.254
DC^fs^, DC^ft^, DC^st^	0.009
DC^fst^	0.009

**Table 4.**
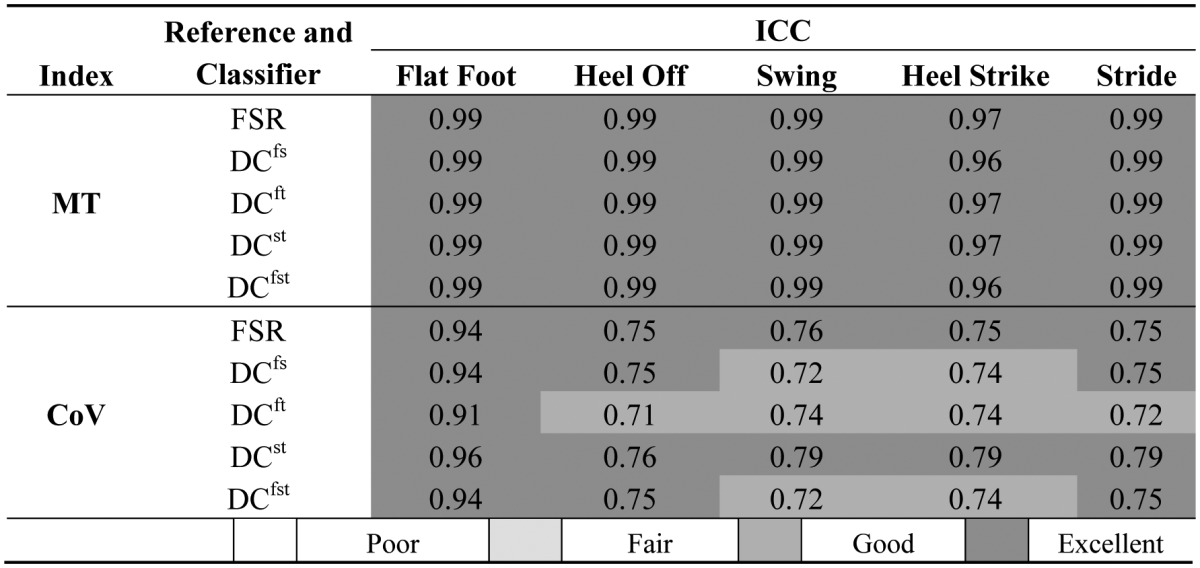
Values of ICC for Mean Time (*MT*) and Coefficient of Variation (*CoV*) evaluated for stride and each gait phase for FSR and DCs.

## References

[1] Simon S.R. (2004). Quantification of human motion: Gait analysis-benefits and limitations to its application to clinical problems. J. Biomech..

[2] Wren T.A., Gorton G.E., Ounpuu S., Tucker C.A. (2011). Efficacy of clinical gait analysis: A systematic review. Gait Posture.

[3] Ryoo M.S., Aggarwal J.K. Hierarchical recognition of human activities interacting with objects.

[4] Leutheuser H., Schuldhaus D., Eskofier B.M. (2013). Hierarchical, multi-sensor based classification of daily life activities: Comparison with state-of-the-art algorithms using a benchmark dataset. PLoS One.

[5] Abaid N., Cappa P., Palermo E., Petrarca M., Porfiri M. (2013). Gait detection in children with and without hemiplegia using single-axis wearable gyroscopes. PLoS One.

[6] O'Connor C.M., Thorpe S.K., O'Malley M.J., Vaughan C.L. (2007). Automatic detection of gait events using kinematic data. Gait Posture.

[7] Zeni J., Richards J., Higginson J.S. (2008). Two simple methods for determining gait events during treadmill and overground walking using kinematic data. Gait Posture.

[8] Desailly E., Daniel Y., Sardain P., Lacouture P. (2009). Foot contact event detection using kinematic data in cerebral palsy children and normal adults gait. Gait Posture.

[9] Miller A. (2009). Gait event detection using a multilayer neural network. Gait Posture.

[10] Boulgouris N.V., Huang X. (2013). Gait recognition using HMMs and dual discriminative observations for sub-dynamics analysis. IEEE Trans. Image Process..

[11] Bamberg S.J.M., Benbasat A.Y., Scarborough D.M., Krebs D.E., Paradiso J.A., Member S. (2008). Gait analysis using a shoe-integrated wireless sensor system. IEEE Trans. Inf. Technol. Biomed..

[12] Goršič M., Kamnik R., Ambrožič L., Vitiello N., Lefeber D., Pasquini G., Munih M. (2014). Online phase detection using wearable sensors for walking with a robotic prosthesis. Sensors.

[13] Bae J., Tomizuka M. (2011). Gait phase analysis based on a Hidden Markov Model. Mechatronics.

[14] Preece S.J., Kenney L.P.J., Major M.J., Dias T., Lay E., Fernandes B.T. (2011). Automatic identification of gait events using an instrumented sock. J. Neuroeng. Rehabil..

[15] Sun H., Yuao T. (2012). Curve aligning approach for gait authentication based on a wearable accelerometer. Physiol. Meas..

[16] Mannini A., Sabatini A.M. (2012). Gait phase detection and discrimination between walking-jogging activities using hidden Markov models applied to foot motion data from a gyroscope. Gait Posture.

[17] Formento P.C., Acevedo R., Ghoussayni S., Ewins D. (2014). Gait event detection during stair walking using a rate gyroscope. Sensors.

[18] Hundza S., Hook W., Harris C. (2013). Accurate and reliable gait cycle detection in Parkinson's disease. IEEE Trans. Neural Syst. Rehabil. Eng..

[19] Nogueira S.L., Siqueira A.G., Inoue R.S., Terra M.H. (2014). Markov jump linear systems-based position estimation for lower limb exoskeletons. Sensors.

[20] Blaya J.A., Herr H. (2004). Adaptive control of a variable-impedance ankle-foot orthosis to assist drop-foot gait. IEEE Trans. Neural Syst. Rehabil. Eng..

[21] Furusho J., Kikuchi T., Tokuda M., Kakehashi T., Ikeda K., Morimoto S., Hashimoto Y., Tomiyama H., Nakagawa A., Akazawa Y. Development of shear type compact MR brake for the intelligent ankle-foot orthosis and its control.

[22] Kanthi M., Karteek I.S.V., Mruthyunjaya H.S., George V.I. Real-time control of active ankle foot orthosis using LabVIEW and Compact-RIO.

[23] Cheng W.-C., Jhan D.-M. (2013). Triaxial Accelerometer-Based Fall Detection Method Using a Self-Constructing. IEEE J. Biomed. Heal. Inf..

[24] Li S., Wang J., Wang X. A novel gait recognition analysis system based on body sensor networks for patients with parkinson's disease.

[25] Chu J.-U., Song K.-I., Han S., Lee S.H., Kang J.Y., Hwang D., Suh J.-K.F., Choi K., Youn I. (2013). Gait phase detection from sciatic nerve recordings in functional electrical stimulation systems for foot drop correction. Physiol. Meas..

[26] Mannini A., Sabatini A.M. (2010). Machine learning methods for classifying human physical activity from on-body accelerometers. Sensors.

[27] Kolawole A., Tavakkoli A. (2012). A novel gait recognition system based on Hidden Markov Models. Adv. Vis. Comput. Lect. Notes Comput. Sci..

[28] Banos O., Damas M., Pomares H., Rojas F., Delgado-Marquez B., Valenzuela O. (2013). Human activity recognition based on a sensor weighting hierarchical classifier. Soft Comput..

[29] Zappi P., Stiefmeier T., Farella E., Roggen D., Benini L., Tr G. Activity recognition from on-body sensors by classifier fusion: Sensor scalability and robustness.

[30] Kittler J., Society I.C., Hatef M., Duin R.P.W., Matas J. (1998). On combining classifiers. IEEE Trans. Pattern Anal. Mach. Intell..

[31] Doyle S., Rodriguez C., Madabhushi A., Tomaszeweski J., Feldman M. Detecting prostatic adenocarcinoma from digitized histology using a multi-scalehierarchical classification approach.

[32] Marin M., Sucar L.E., Gonzalez J.A., Diaz R. A hierarchical model for morphological galaxy classification.

[33] Zhao X., Li M., Song G., Xu J. (2010). Hierarchical ensemble-based data fusion for structural health monitoring. Smart Mater. Struct..

[34] Lin C., Zou Y., Qin J., Liu X., Jiang Y., Ke C., Zou Q. (2013). Hierarchical classification of protein folds using a novel ensemble classifier. PLoS One.

[35] Voisin A., Krylov V.A., Moser G., Serpico S.B. (2013). Classification of very high resolution SAR images of urban areas using copulas and texture in a hierarchical Markov random field model. IEEE Geosci. Remote Sens. Lett..

[36] Rabineer L. (1989). A tutorial on Hidden Markov Models and selected applications in speech recognition. Proc. IEEE.

[37] Rabiner L., Juang B.H. (1986). An introduction to hidden Markov models. IEEE ASSP Mag..

[38] Jordan M.I. (2004). Graphical models. Stat. Sci..

[39] Palermo E., Rossi S., Patanè F., Cappa P. (2014). Experimental evaluation of indoor magnetic distortion effects on gait analysis performed with wearable inertial sensors. Physiol. Meas..

[40] Favre J., Aissaoui R., Jolles B.M., de Guise J.A., Aminian K. (2009). Functional calibration procedure for 3D knee joint angle description using inertial sensors. J. Biomech..

[41] Cutti A., Ferrari A., Garofalo P., Raggi M., Cappello A., Ferrari A. (2010). “Outwalk”: A protocol for clinical gait analysis based on inertial and magnetic sensors. Med. Biol. Eng. Comput..

[42] Palermo E., Rossi S., Marini F., Patanè F., Cappa P. (2014). Experimental evaluation of accuracy and repeatability of a novel body-to-sensor calibration procedure for inertial sensor-based gait analysis. Measurement.

[43] Ainsworth B.E., Haskell W.L., Whitt M.C., Irwin M.L., Swartz M., Strath S.J., O'Brien W.L., Bassett D.R., Schmitz K.H., Emplaincourt P.O. (2000). Compendium of physical activities: An update of activity codes and MET intensities. Med. Sci. Sports Exerc..

[44] Cawley G.C., Talbot N.L.C. (2003). Efficient leave-one-out cross-validation of kernel fisher discriminant classifiers. Pattern Recognit..

[45] Aung M.S.H., Thies S.B., Kenney L.P.J., Howard D., Selles R.W., Findlow A.H., Goulermas J.Y. (2013). Automated detection of instantaneous gait events using time frequency analysis and manifold embedding. IEEE Trans. Neural Syst. Rehabil. Eng..

[46] Hausdorff J.M., Zemany L., Peng C., Goldberger A.L. (1999). Maturation of gait dynamics: Stride-to-stride variability and its temporal organization in children. Appl. Physiol..

[47] Cicchetti D.V. (1994). Guidelines, criteria, and rules of thumb for evaluating normed and standardized assessment instruments in psychology. Psychol. Assess..

[48] Tanida S., Kikuchi T., Otsuki K., Ozawa T., Fujikawa T., Yasuda T., Furusho J., Shoji A., Hashimoto Y. Intelligently controllable Ankle Foot Orthosis (I-AFO) and its application for a patient of Guillain-Barre syndrome.

[49] Shorter A.K., Kogler G.F., Loth E., Durfee W.K., Hsiao-Wecksler E.T. (2011). A portable powered ankle-foot orthosis for rehabilitation. J. Rehabil. Res. Dev..

[50] Krebs H.I., Rossi S., Kim S.-J., Artemiadis P.K., Williams D., Castelli E., Cappa P. Pediatric anklebot.

[51] Weinberg B.A., Nikitczuk J.A., Patel S.A., Patritti B., Mavroidis C., Bonato P., Canavan P. Design, control and human testing of an active knee rehabilitation orthotic device.

[52] Nikitczuk J.A., Weinberg B.A., Canavan P., Mavroidis C. (2010). Active knee rehabilitation orthotic device with variable damping characteristics implemented via an electrorheological fluid. IEEE/ASME Trans. Mechatron..

[53] Lemaire E.D., Goudreau L., Yakimovich T., Kofman J. (2009). Angular-velocity control approach for stance-control orthoses. IEEE Trans. Neural Syst. Rehabil. Eng..

[54] Brisswalter J., Mottet D. (1996). Energy cost and stride duration variabilitu at preferred transition gait speed between walking and running. Appl. Physiol..

[55] Liu Y., Lu K., Yan S., Sun M., Lester D.K., Zhang K. (2014). Gait phase varies over velocities. Gait Posture.

[56] Riva F., Bisi M.C., Stagni R. (2014). Gait variability and stability measures: Minimum number of strides and within-session reliability. Comput. Biol. Med..

